# Lactate Dehydrogenase 5 Expression in Non-Hodgkin Lymphoma Is Associated with the Induced Hypoxia Regulated Protein and Poor Prognosis

**DOI:** 10.1371/journal.pone.0074853

**Published:** 2013-09-23

**Authors:** Renquan Lu, Minglei Jiang, Zhujun Chen, Xiaofeng Xu, Hongfeng Hu, Xinmin Zhao, Xiang Gao, Lin Guo

**Affiliations:** 1 Department of Clinical Laboratory, Shanghai Cancer Center, Fudan University, Shanghai, China; 2 Department of Oncology, Shanghai Medical College, Fudan University, Shanghai, China; 3 Department of Medical Oncology, Shanghai Cancer Center, Fudan University, Shanghai, China; Institut Pasteur of Shanghai, Chinese Academy of Sciences, China

## Abstract

Lactate dehydrogenase 5 (LDH-5) is one of the major isoenzymes catalyzing the biochemical process of pyruvate to lactate. The purpose of this study was to investigate the expression of serum LDH-5 and test whether this enzyme is regulated by tumor hypoxia and represents a prognostic marker in patients with Non-Hodgkin’s lymphoma (NHL). In this study, LDH-5 levels were detected using agarose gel electrophoresis in NHL patients (n = 266) and non-NHL controls including benign lymphadenectasis (n = 30) and healthy cohorts (n = 233). We also explored the expression of LDH-5 and hypoxia-inducible factor (HIF) 1α in NHL and benign controls by immunohistochemistry and immunofluorescence staining, respectively. Moreover, the role of LDH-5 in the progression of NHL was assessed by multivariate Cox analyses and Kaplan-Meier survival estimates. Serum concentrations of LDH-5 were significantly higher in NHL patients (9.3%) than in benign patients and healthy controls (7.5% and 7.2%, respectively, *P*<0.01). Application of LDH-5 detection increased the sensitivity of NHL detection, identifying 53.4% of NHL patients as positive, compared with the measurement of total LDH levels (36.5% sensitivity). LDH-5 concentrations increased with clinical stage, extra-nodal site involvement, and WHO performance status of patients with NHL. Exposure to a hypoxic environment induced the expression of LDH-5 and its overexpression correlated with HIF1α cytoplasmic accumulation in NHL cells. In multivariate analyses, LDH-5 was an independent marker for progression-free survival in patients with NHL (*P*<0.001). Overall, the expression of LDH-5 was elevated in NHL, showing an association with tumor hypoxia and unfavorable prognosis. Thus, LDH-5 emerges as a promising prognostic predictor for NHL patients.

## Introduction

Much effort has been devoted to identifying potential biomarkers for cancer in recent years [1]–[2]. Among these markers, enzymes play important roles in energy metabolism of cancer cells and participate avidly in cell proliferation and the growth process [3].

It is well known that glycolysis is a major pathway for energy acquisition. Cancer cells prefer the anaerobic pathway of glycolysis despite the presence of oxygen [4]–[6]. Tumor cells actively metabolize carbohydrates to produce lactic acid rather than catabolizing glucose via the tricarboxylic acid cycle. Thus, up-regulation of lactate dehydrogenase (LDH) in cancer cells ensures efficient glycolytic metabolism in tumors and reduced dependence on oxygen [7]. Furthermore, tumors display glycolysis driven by hypoxia and/or oncogenic mutations, with the pyruvate to lactate conversion being promoted by increased expression of LDH. This is simultaneously enhanced by stabilization of the hypoxia-inducible factor (HIF) during the adaptive response to the hypoxic tumor microenvironment [8].

**Figure 1:**
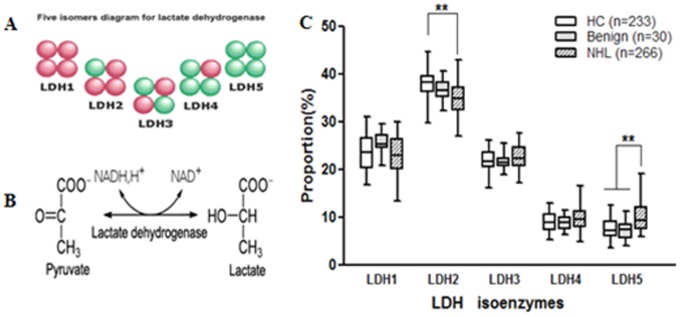
Comparison of expression levels of the five LDH isoenzymes. (A) Schematic depicting the five isomers of LDH, indicating subunit composition. (B) LDH enzymes catalyze the metabolism of pyruvate to lactate to acquire the energy in the glycolytic pathway. (C) Expression levels of the five LDH isoenzymes in the serum of patients with NHL (NHL) and benign (Benign) or healthy controls (HC). **, *P*<0.01.

The actions of LDH and its isoenzymes have been suggested to be valuable markers in identifying different kinds of malignancies, and may be especially useful as adverse prognostic factors in poor-risk carcinomas [9], [10]. There are five isoenzymes of LDH comprised of different subunits, with LDH-1 containing four H-subunits, while LDH-5 consists of four M-subunits. Pyruvate is converted to lactate by LDH isoenzymes under hypoxic conditions whereby adenosine triphosphate (ATP) and nicotinamide adenine dinucleotide are generated (Figure 1A and 1B). With increasing number of M- over H-chains, LDH-5 becomes the most efficient isoenzyme in catalyzing the conversion of pyruvate to lactate [11]. LDH-5 is therefore an important isoenzyme under hypoxic conditions and may play a vital role in the development and progression of malignancies.

As demonstrated previously, LDH-5 in serum is a potential biomarker for various malignancies such as leukemia, gastric and, endometrial cancers [12]–[14]. Acidification of the extracellular matrix by lactate release is probably one of the main factors triggering aggressive behavior in such tumors. More than half of lung and colorectal adenocarcinomas overexpress LDH-5 and this is linked with aggressive phenotype and unfavorable prognosis [15], [16]. However, the role of LDH-5 in Non-Hodgkin’s lymphoma (NHL) has not, to the best of our knowledge, been clearly described. In this study, the expression of LDH isoenzymes was measured in patients with NHL and compared with benign and heath controls. Meanwhile, the expression of LDH-5, the major LDH isoenzyme responsible for sustaining anaerobic glycolysis, was examined in the tissues of NHL patients and compared with HIF1α expression. Additionally, we have also investigated whether LDH-5 expression is an independent marker of unfavorable prognosis in patients with malignant NHL.

## Materials and Methods

### Study Population and Peripheral Blood Samples

We recruited adult patients with NHL to a test cohort from the Department of Oncology, Fudan University Shanghai Cancer Center, Shanghai, China, from September 2006 to December 2012. Of the 266 patients recruited, 115 were female (43%), with median age at diagnosis of 51 years (range, 21–81 years), and 151 patients were male (57%), the median age at diagnosis was 49 years (range, 18–78 years). All diagnoses of NHL were based on histological biopsy and were made according to the updated Working Formulation Scheme [17]. Clinicopathological characteristics are shown in Table 1. LDH-5 levels were determined at time of follow-up in all NHL patients with chemotherapy, under the R-CHOP or R-CEOP guidelines. All 266 patients were diagnosed with malignant lymphoma by a pathologist with a special interest in lymphoma classification. The histologic lymphoma types consisted of small lymphocytic cell lymphoma (3 patients; 1.1%); B cell lymphocytic lymphoma (3 patients; 1.1%); diffuse large B cell lymphoma (DLBCL; 179 patients; 67.3%); follicular lymphoma (FL; 27 patients; 10.1%); NK/T cell lymphoma (26 patients; 9.8%); anaplastic large cell lymphoma (5 patients; 1.9%); Burkitt’s lymphoma (5 patients; 1.9%); FL, mixed large B cell lymphoma (10 patients; 3.8%); FL, mixed small and large cell lymphoma (3 patients; 1.1%); angioimmunoblastic T cell lymphoma (2 patients; 0.8%); and other lymphomas (3 patients; 1.1%). All patients were followed regularly at intervals of 3 months in an outpatient department. The median follow-up for the patients was 50 months (range, 28–120 months).

**Table 1:** LDH-5 levels and the clinicopathological characteristics of 266 patients with NHL.

		LDH-5 Levels		
Cases	LDH-5Levels	Positivecases(%)	Negativecases(%)	*P* value
Gender				
Female	9.3 (7.7–11.2)	61 (53.0)	54 (47.0)	
Male	9.3 (7.3–12.2)	81 (53.6)	70 (46.4)	1.000
Age				
≤60	9.4 (7.3–11.8)	112 (56.3)	87 (43.7)	
>60	9.0 (8.1–12.6)	30 (44.8)	37 (55.2)	0.120
Histological type				
DLBCL	9.0 (7.2–11.2)	87 (48.6)	92 (51.4)	
FL	9.9 (8.3–12.4)	19 (70.4)	8 (29.6)	0.040
NK/T	9.6 (7.6–12.1)	14 (53.8)	12 (46.2)	0.678, 0.264*
Mixed	11.1 (8.6–12.4)	9 (69.2)	4 (30.8)	0.250, 1.000*
ALCL	11.0 (7.5–16.8)	3 (60.0)	2 (40.0)	0.677, 0.637*
Burkitt’s	12.2 (7.9–14.1)	3 (60.0)	2 (40.0)	0.677, 0.637*
Others	11.9 (7.8–12.7)	7 (63.6)	4 (36.4)	0.369, 0.714*
Total LDH level				
<250	9.3 (7.4–11.6)	93 (54.1)	79 (45.9)	
≥250	9.4 (7.7–12.7)	49 (52.1)	45 (47.9)	0.798
Clinical stage				
I	8.3 (7.2–9.6)	17 (35.4)	31 (64.6)	
II	8.5 (7.0–9.9)	37 (38.5)	59 (61.5)	0.855
III	10.9 (8.1–13.4)	58 (68.2)	27 (31.8)	<0.001
IV	12.3 (9.6–16.1)	30 (81.1)	7 (18.9)	<0.001
Extra-nodal site(s) involvement				
<2	8.4 (7.0–9.9)	78 (39.6)	119 (60.4)	
≥2	13.4 (12.2–17.0)	64 (92.7)	5 (7.3)	<0.001
WHO performance status				
0–1	8.6 (7.1–10.0)	85 (42.5)	115 (57.5)	
2–4	13.4 (11.1–17.1)	57 (86.4)	9 (13.6)	<0.001

* compared with follicular lymphoma.

We also recruited patients with benign lymphadenectasis (n = 30; age range, 27–79 years) and healthy controls (n = 233; age range, 20–79 years) from the outpatients department, Fudan University Shanghai Cancer Center, Shanghai, China.

Five milliliter of peripheral venous blood was collected in a sterile test tube at the time of the diagnosis and before the start of lymphoma treatment. Blood samples were centrifuged at 3000×g for 10 minutes, then separated and stored at −80°C until they were analyzed. The study design was approved by the Clinical Research Ethics Committee of the Fudan University Shanghai Cancer Center. All of the participants signed informed consent documents prior to participating in this study.

### Quantitative Analysis of Serum LDH Isoenzymes

The LDH isoenzyme levels were detected by agarose electrophoresis using the SPIFE 3000 systems and SPIFE® LD Isoenzyme (Helena Laboratories Inc), according to the manufacturer’s instructions. Briefly, SPIFE 3000 was performed as follows: 80 µL of NHL patient or the control serum was collected. After electrophoresis separation, each isoenzyme was detected colorimetrically by scanning the LDH isoenzyme gel and analyzed by Platinum 3.0 software.

### Human Tissue Specimens from NHL Patients and Benign Lymphadenectasis Patients

Formalin-fixed paraffin-embedded tissues from 20 patients with stage I–IV NHL (aged 20–58 years) and eight samples from control patients with benign lymphadenectasis (aged 18–60 years) were retrieved from the department of pathology (between July 2009 and December 2012), Fudan University Shanghai Cancer Center. All cases were confirmed by pathology. Use of this tissue was approved by the Institute Review Ethics committee. All patients provided their written informed consent documents before inclusion.

### Tissue Immunohistochemistry

Immunohistochemistry (IHC) staining for LDH-5 and HIF1α was performed on formalin-fixed paraffin-embedded tissues using a protocol as previously described [18]. LDH-5 was detected using a commercially available rabbit polyclonal antibody (Abcam, Cambridge, MA, USA) at a dilution of 1∶200. To evaluate HIF1α expression, we used mouse monoclonal antibody MAb458400 (dilution 1∶500, Invitrogen, Camarillo, CA, USA). IHC staining was assessed by two independent pathologists under blinded conditions and any discrepancies were resolved by consensus. The concordance between scores from different cores of the same tumor was greater than 90%. The intensity of LDH-5 staining was evaluated using the following criteria: strong staining (score 3), dark brown staining in >50% of cells completely obscuring cytoplasm; medium staining (score 2), any lesser degree of brown staining appreciable in 10%–50% cell cytoplasm; weak staining (score 1), yellow staining in <10% cell cytoplasm; and negative (score 0), no appreciable staining in any cell [14]. For HIF1α protein immunoreaction, we regarded >50% of cells exhibiting strong cytoplasmic and/or >10% of cells with nuclear staining as a positive result, whereas weak cytoplasmic expression (to any extent) or strong cytoplasmic expression in <50% of cells was considered as low expression [19], [20].

### Immunofluorescence Staining

The expression of LDH-5 and HIF1α was assessed by immunofluorescence (IF) as previously described [21], [22]. Briefly, three-micron tissue sections were fixed by immersion in freshly prepared methanol/acetone (1∶1, v/v) for 10 minutes at −20°C. Sections were then incubated overnight at 4°C with 1∶100 rabbit anti-LDH-5 antibody (Abcam, Cambridge, MA, USA) or 1∶200 mouse anti-HIF1α antibody (Invitrogen, Camarillo, CA, USA) at room temperature for 90 minutes. After rinsing three times with TBS, sections were treated with Alexa Fluor 488 or 592- conjugated secondary antibodies (Santa Cruz Biotechnology Inc., Santa Cruz, CA, USA), and finally viewed by confocal microscopy.

### HIF1α Immunoassays

Serum levels of HIF1α in patients with NHL or the control cohort were determined using a commercial enzyme-linked immunosorbent assay (USCN Life Science Inc., Houston, TX, USA) as described by the manufacturer. In brief, the samples were added to wells, and then incubated for 2 hours at 37°C. Detection reagent A (biotin-conjugated anti-HIF1α antibody) was added to each well, followed by detection reagent B (Horseradish Peroxidase (HRP)-conjugated avidin), and substrate solution after washing at each step. Finally stop solution was added and plates were read at 450 nm.

### Statistical Analysis

Statistical analyses were performed using SPSS13.0 software (SPSS Inc., Chicago, IN). The data were expressed as median value (interquartile range, Q1–Q3), with nonparametric analyses used to assess differences. Kruskal-Wallis analysis of variance was used for comparison between groups. Receiver operating characteristic (ROC) curve analysis was used to quantify LDH-5 performance. Linear regression analysis was employed to assess correlation with continuous variables. A Cox proportional hazards model was used to assess the effect of parameters on progress events. Progression-free survival (PFS) time was computed by the Kaplan–Meier method and analyzed with the log-rank test. A *P* value of less than 0.05 was considered statistically significant.

## Results

### Comparison of LDH-5 Levels between NHL Patients and Non-NHL Controls

To examine the expression and level of the five LDH isoenzymes in NHL, serum samples from 529 subjects (266 NHL patients, 30 benign lymphadenectasis and 233 healthy controls) were determined by agarose electrophoresis assay (Figure 1). The cohorts were closely matched for sex, but fewer senior patients (age >60) with NHL were enrolled into the test cohort. As shown in Figure 1C, the pattern of LDH isoenzyme distribution in serum differed between NHL patients and benign/healthy controls, which may be helpful in identifying a cohort of malignant patients. Specifically, serum concentrations of LDH-5 were significantly higher in NHL patients (9.3%; range 7.5%–11.9%) than those of benign patients and healthy controls (7.5%; range 5.6%–8.5% and 7.2%; range 5.8%–9.1%, respectively; *P*<0.01). Conversely, LDH-2 expression was reduced in patients with NHL compared with the control cohort (Figure 1C), while LDH-1, LDH-3 and LDH-4 values did not differ significantly between the three groups.

By applying the updated Working Formulation classification of NHL, the present study demonstrated that LDH-5 levels were higher in patients with all kinds of NHL than those of Non-NHL controls (benign and healthy participants), especially in patients with FL and Burkitt’s lymphoma. FL had a significantly higher LDH-5 concentration than DLBCL (Table 1; *P*<0.05).

**Figure 2:**
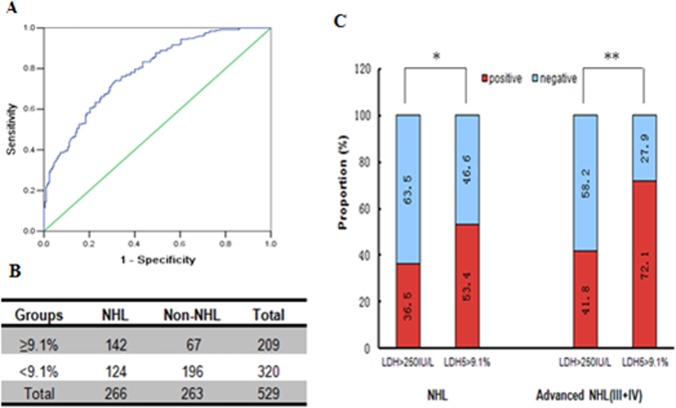
Comparison of LDH-5 and total LDH levels in NHL patient serum. (A) ROC analysis of LDH-5 showed that the cutoff value set at 9.1% gives the best diagnostic efficiency (AUC = 0.788, n = 529). (B) The sensitivity and specificity for NHL diagnosis were 53.4% (142/266) and 74.6% (196/263) at the LDH-5 cutoff value (9.1%), respectively. (C) Serum LDH-5 concentration ≥9.1% has more positive results than total LDH ≥250 IU/L in all NHL patients (n = 266) and patients with advanced NHL (III+IV, n = 122). *P<0.05; **P<0.01.

Moreover, the ROC curves indicated the optimum diagnostic cutoff value for LDH-5 was 9.1% (AUC = 0.788, 95%CI = 0.747–0.828), shown in Figure 2. The sensitivity and specificity for the diagnosis of NHL were 53.4% (142/266) and 74.6% (196/263), respectively, while the cutoff value of LDH-5 level was set at 9.1%. In addition, positive and negative predictive values for LDH-5 in the identification of the patients with NHL are shown as 68.0% (142/209) and 61.3% (196/320), respectively. When we chose 250 IU/L as the cutoff value for LDH in this study, a greater proportion of patients with NHL in the test cohort were positive for LDH-5 than for total LDH (142 [53.4%] vs 97 [36.5%] of 266 patients, *P*<0.05). Furthermore, 88 of 122 patients (72.1%) with advanced NHL had positive LDH-5 results, whereas only 51 (41.8%) of those showed total LDH positivity (Figure 2, *P*<0.01). Therefore, the raised serum concentrations of LDH-5 could be used to more easily distinguish patients with NHL.

### LDH-5 Level and the Clinicopathological Characteristics of Patients with NHL

Since patients with NHL had significantly higher levels of LDH-5 than total LDH, we further investigated the relationship between the clinicopathological characteristics of NHL patients and serum levels of LDH-5. In the test cohort, 144 of 266 patients (54.1%) with NHL had early stage disease (clinical stage I+II), while the remainder (45.9%) had advanced stage (clinical stage III+IV). Serum LDH-5 concentration improved differential diagnosis of advanced stage NHL from patients with early stage NHL (*P*<0.001, Table 1). As shown in Table 1, LDH-5 levels also increased significantly with extra-nodal site involvement (*P*<0.001), and WHO performance status (*P*<0.001).

### LDH-5 and HIF1α Expression Were Analyzed by Immunohistochemistry

**Figure 3:**
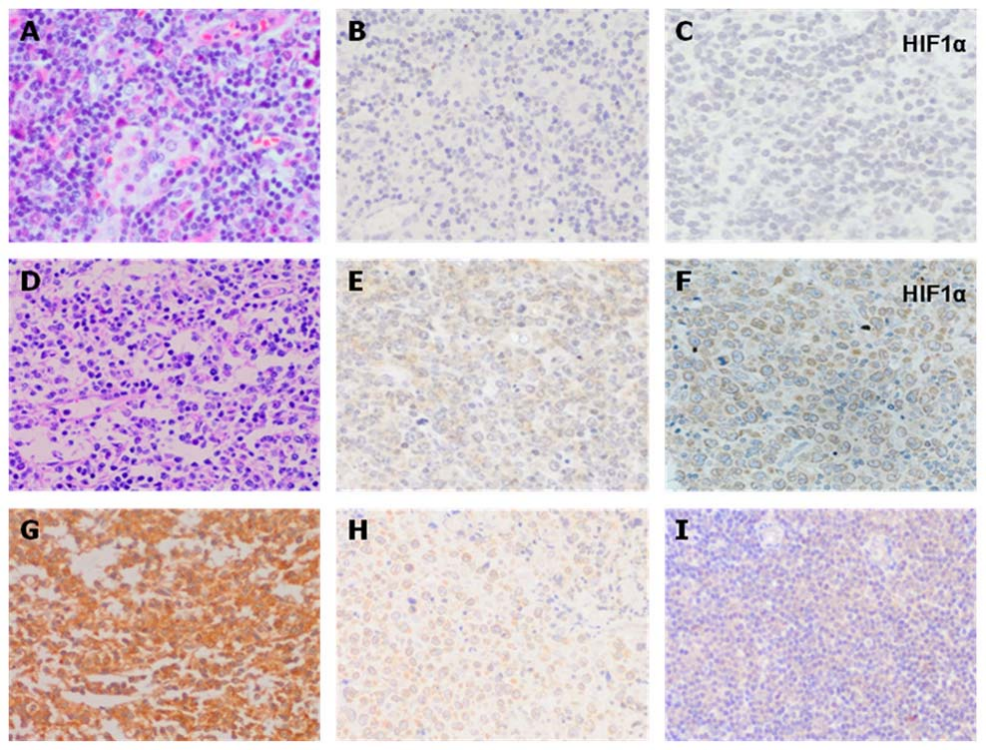
Expression patterns of LDH-5 and HIF1α shown by immunohisto-chemical staining in patient tissues. (A–C) Patients with benign lymphadenectasis, (A) representative examples of HE staining, (B) LDH-5 expression (negative), and (C) HIF1α expression (weak positive); (D–I) Patients with NHL, (D) HE staining; (E) LDH-5 expression in DLBCL (positive), (F) HIF1α expression in DLBCL (positive), and (G) strongly positive expression of LDH-5 in follicular lymphoma; (H) strongly positive expression of LDH-5 in NK/T cell lymphoma; (I) positive expression of LDH-5 in small cell lymphoma. Original magnification, ×400.

We next explored the relationship between HIF1α and LDH-5 expression in clinical tissue samples from patients with NHL (*n = *20) and lymphadenectasis (*n* = 8) by IHC staining. Representative images for LDH-5 and HIF1α are presented in Figure 3. Cytoplasmic/nuclear staining of HIF1α was observed in 10 of the 28 tissue samples (35.7%), membranous staining in eight cases (28.6%), and no staining in 10 cases (35.7%). As for HIF1α, 16 of 20 cases (80%) in the NHL group and two of eight (25%) lymphadenectasis patients showed positive staining. LDH-5 staining with a polyclonal antibody specific for LDH-5 was mainly observed in the cytoplasm of tumor cells. Cytoplasmic staining was scored as negative, weak, moderate or strong; nuclear expression, when present, was accompanied with moderate/strong cytoplasmic reactivity, although pure nuclear expression was occasionally noted. Tumors were scored in a four-scale system according to the intensity and extent of staining, LDH-5 was strongly detectable in five of the 20 samples (25.0%), of medium intensity in 12 cases (60.0%) and stained only weakly in 3 cases (15.0%). Conversely, LDH-5 expression was shown to be either negative (75.0%) or weak (25.0%) by IHC staining in the tissues of patients with benign lymphadenectasis (Table 2).

**Table 2:** Grading system based on the intensity and extent of LDH5 cytoplasmic and nuclear staining.

Scoring system Subjects	Negative(0)	Weak(1)	Medium(2)	Strong(3)
Lymphadenectasis	6 (75.0)	2 (25.0)	0 (0.0)	0 (0.0)
B cell NHL				
DLBCL	0 (0.0)	1 (5.0)	5 (25.0)	1 (5.0)
FL	0 (0.0)	0 (0.0)	2 (10.0)	3 (15.0)
Small cell lymphoma	0 (0.0)	0 (0.0)	2 (10.0)	0 (0.0)
T cell NHL				
NK/T cell lymphoma	0 (0.0)	1 (5.0)	2 (10.0)	1 (5.0)
Mixed T cell lymphoma	0 (0.0)	1 (5.0)	1 (5.0)	0 (0.0)

### LDH-5 and HIF1α Expression Were Analyzed by Immunofluorescence Staining

**Figure 4:**
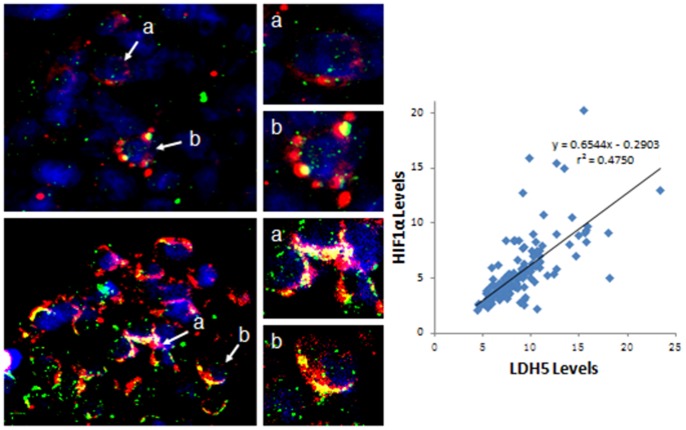
Immunofluorescence staining for the expression of LDH-5 and HIF1α in malignant NHL and benign lymphadenectasis tissues. (A) Colocalization of LDH-5 (green) and HIF1α (red) in benign lymphadenectasis (nuclear staining, blue); (B) Colocalization of LDH-5 (green) and HIF1α (red) in malignant NHL (nuclear staining, blue); (C) The correlation of serum LDH-5 and HIF1α expression in peripheral blood of the participants (*n* = 160) including the patients with NHL (*n* = 130) and Non-NHL controls (*n* = 30).

As shown in Figure 3, HIF1α has a different expression pattern in lymphadenectasis (weak positive, Figure 3C) and DLBCL (positive, Figure 3F) using IHC. We further showed by IF staining that a similar LDH-5 expression pattern exists in benign lymphadenectasis (*n = *8) and malignant NHL (DLBCL, *n = *7) (positive, *n* = 3 and 5, respectively; Figure 4A and 4B, LDH-5 staining green). Furthermore, malignant transformation induced the expression of LDH-5 and its colocalization with HIF1α (Figure 4B, yellow indicates colocalization) in DLBCL cells, while only limited colocalization was observed in benign disease, although HIF1α was expressed to some extent (Figure 4A, HIF1α staining red).

In addition, serum LDH-5 concentration is abnormal (≥9.1%) in patients with NHL and a few Non-NHL controls. This correlates with HIF1α expression (*n = *160, r^2^ = 0.4750; *P*<0.01, Figure 4C) and is a mechanism of regulating the hypoxic environment created by rapid tumor growth. As LDH-5 levels increase with disease progression, LDH-5 expression may be a useful prognostic factor in NHL patients.

### Prognostic Significance Assessed by Multivariate and Kaplan-Meier Progression-free Survival Analysis

**Figure 5:**
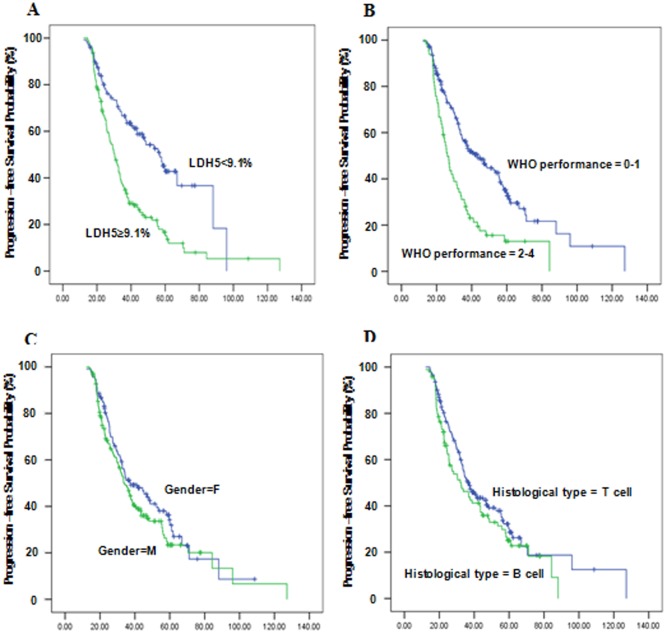
Kaplan-Meier progression-free survival curve associated with serum LDH-5 concentrations in 266 NHL patients. (A) The PFS probability of patients with elevated serum LDH-5 concentrations (>9.1%) was significantly lower than that of patients with normal concentrations (log-rank test, *P*<0.001); (B) The worse WHO performance status (2–4) was also a significant unfavourable predictor for PFS (*P*<0.001); (C, D) The PFS was not associated with the gender and histological type of patients (*P = *0.199 and *P = *0.153, respectively). PFS, progression-free survival.

We further investigated the role of LDH-5 in the prognosis of NHL patients. For the whole study population, the PFS rates were 59.8% and 38.7% at 3 and 5 years, respectively. Multivariate analysis using a Cox proportional hazard model showed that clinical performance and LDH-5 concentration were significant unfavorable predictors for PFS (*P*<0.05, Table 3). In addition, there was a tendency for a correlation between clinical stage and PFS (*P = *0.063; Table 3). Kaplan-Meier PFS and log-rank analysis further demonstrated that LDH-5 was an independent prognostic marker for PFS in NHL patients (*P*<0.001, Figure 5A); the PFS probability of the patients with worse clinical performance status (2–4) was significant lower than that of patients with the performance status 0 or 1 (*P*<0.001, Figure 5B); however, there was almost no correlation between PFS probability and the gender/histological type of patients (*P = *0.199 and *P = *0.153, respectively, Figure 5C and 5D).

**Table 3:** Multivariate Cox proportional hazard analyses.

Variables	Hazard ratio (95% CI)	*P* Value
Gender (F vs M)	1.137 (0.830–1.556)	0.423
Age (≤60 vs >60)	0.906 (0.630–1.305)	0.597
Clinical stage (I, II vs III, IV)	1.368 (0.983–1.904)	0.063
Histological type (T cell vs B cell)	1.149 (0.728–1.814)	0.550
LDH (<250 vs ≥250)	1.061 (0.759–1.483)	0.729
Extra-nodal (<2 vs ≥2)	1.129 (0.684–1.863)	0.636
WHO performance status(0–1 vs 2–4)	1.576 (1.112–2.234)	0.011
M/H proportion(<0.547 vs >0.547)	1.164 (0.783–1.731)	0.454
LDH-5 concentration(normal vs elevated)	1.704 (1.146–2.533)	<0.001

## Discussion

NHL is a group of cancers derived from lymphocytes and includes any kind of lymphoma except Hodgkin’s lymphoma. In 2012, NHL was the seventh most common malignant disease and the ninth leading cause of cancer-related death worldwide [23]. Although advances in noninvasive biomarker detection have improved our ability to manage many malignancies, the currently available markers for NHL are still scarce or unsatisfactory, with low sensitivity in detecting the presence of cancer cells. Therefore, there is an urgent need to identify reliable biomarkers for clinical use. Specifically, the application of serum markers in NHL management includes aiding diagnosis, monitoring response or resistance to therapy, and prospectively predicting disease prognosis.

Under the anaerobic microenvironment in NHL, pyruvate produces lactic acid, providing energy rapidly in the form of ATP. LDH and its isoenzymes play an important role in this process. In the main subtypes of NHL, including DLBCL, FL and other subgroups, increased serum LDH levels in patients have been associated with aggressive clinical behavior, resistance to chemotherapy and poor survival [24], [25]. Similarly, LDH-5 in serum was also used as a criterion for patient stratification during clinical trials for these malignancies [26]. In this study, we showed by agarose gel electrophoresis that serum LDH-5 levels were significantly increased in NHL patients, while LDH-2 is expressed but down-regulated in serum of these patients, compared with benign and healthy controls. Thus, determination of serum LDH-5 may provide a useful and convenient serologic marker for identifying NHL using the classic technique for isoenzyme activity determination, instead of immunoassay. In brief, the detection procedures of LDH-5 include two steps: first, accurate separation of the LDH component subunits; next, determination of the LDH isoenzyme activities by staining and colorimetric analysis. Interesting, this study demonstrated that there was no significant difference in LDH-5 expression among the different subgroups of NHL studied, except that FL had a higher LDH-5 concentration. Moreover, LDH-5 levels also increased significantly with clinical stage, extra-nodal site involvement, and WHO performance status. To our knowledge, this is the first study that demonstrated a strong association between serum LDH-5 concentrations and aggressive clinicopathological characteristics in human NHL with the use of agarose gel electrophoresis detection.

More significantly, we detected that LDH-5 positivity is markedly higher (53.4%; 142 of 266) than that for total LDH level (36.5%; 97 of 266) in all NHL patients. Furthermore, 88 of 122 patients (72.1%) with advanced NHL had positive LDH-5 results, whereas only 51 of these 122 patients (41.8%) had positive total LDH results. Therefore, elevated serum LDH-5 concentrations could efficiently distinguish patients with NHL.

The M- and H-subunits of LDH are encoded by the LDH-A and LDH-B genes, respectively. Recently, it has been reported that LDH-A expression is increased in the serum of cancer patients and linked with disease progression, thus potentially being of use as a screening marker and specific therapeutic predictor [27], [28]. LDH-5 was shown to be the dominant isomer, with significantly increased LDH-M subunit expression in transformed embryonic cells. Conversely, tumors generated from LDH-M deficient clones showed a significantly reduced progression over time compared with controls [27], [29]. Our data showed that LDH-5 expression is closely related to progression of NHL, being barely detectable in lymphadenectasis but strongly elevated with increasing clinical stage and extra-nodal site involvement.

We further investigated the immunohistochemical expression of LDH-5 in a group of 20 patients with NHL by pathologic diagnosis and group of eight control patients with lymphadenectasis treated with surgery alone. LDH-5 overexpression in NHL cancer cells compared with tissues of benign lymphadenectasis was validated by IHC. Furthermore, IHC analysis also revealed the distinct LDH-5 expression profile of FL compared with DLBCL. We therefore concluded that the LDH-A gene is highly upregulated in NHL, particularly in FL. This is directly related to the active HIF1α pathway [30]. Seventeen of 20 NHL cases (85.0%) displayed LDH-5 expression, with medium or high LDH-5 expression being associated with low lymphocytic response; this may suggest an important role of LDH-5 and, presumably, lactate release in tumor escape from host immunosurveillance. Malignant transition induced the expression of LDH-5 and its co-localization with HIF1α in NHL cells, shown by IF staining. In addition, correlation analysis of LDH-5 with hypoxia protein expression in NHL also showed a strong association between LDH-5 with HIF1α. This association may regulate the hypoxic microenvironment and meet the growth and proliferation requirements of the tumor. Koukourakis et al provided evidence that LDH-5 was significantly associated with HIF1α, and tissue LDH-5 gave important prognostic information in operable colorectal cancer [31]. Langhammer also suggested the use of LDH-5 as a potential therapeutic target for anticancer treatment [28], while other studies have demonstrated that LDH-5 is highly upregulated in NHL, particularly in DLBCL [20]. A greater understanding of the regulatory mechanisms in cancer cell metabolism may open additional avenues.

Studies have shown that activation of the HIF-linked molecular cascade leads to tumor aggressiveness by triggering anaerobic metabolism, while pyruvate increases the amount and activity of HIF1α by interference with proteasomal degradation mechanisms [32], [33]. Here we showed that LDH5 overexpression was accompanied by elevated HIF1α in DLBCL. HIF1α in turn stimulates the expression of LDH-A, thereby preventing the accumulation of pyruvate. This feedback loop implies an intricate balance between the different roles of HIF1α, which may be determined by the cumulative effect of multiple interactions within a cell [34], [35]. Kayser et al confirmed a close link between the two metabolic enzymes and indicated an alteration in glucose metabolism during the process of malignant transformation [35], [36]. The exact regulatory mechanism of the hypoxia molecular cascade in cancer cells is close to elucidation, which will help us to perform targeted therapies resulting in surer outcomes.

The clinical significance of LDH-5 as a predictive biomarker in NHL, however, remains largely unexplored. We therefore illustrated the potential utility of serum LDH-5 as a prognostic marker in NHL patients. Our data further showed that clinical performance and LDH-5 concentration were significant unfavorable predictors for PFS by multivariate analysis using a Cox proportional hazard model. Furthermore, our results also revealed that patients with serum LDH-5 levels >9.1% had significantly shorter PFS than those with lower levels of this marker by log-rank test; moreover, there was no correlation between PFS probability and the gender (or histological type) of patients. Therefore, our study indicated that LDH-5 could be a promising diagnostic marker for tumor malignancy and an independent predictor of NHL prognosis. Thus, LDH-5 may be a valuable biomarker for individualized therapy and optimizing the International Prognostic Index evaluation system in NHL.
